# "Let There Be Light"

**Published:** 1908-08-15

**Authors:** J. F. Goodhart


### Hospital Clinics.

### "LET THERE BE LIGHT."

By J. F. GOODHART, LL.D., M.D., F.R.C.P.

(An Address delivered to the Climatological and Balneological Society on June 19, 1908.)

If ever there was a case of the blind man attempting to lead the blind, I come before you to-day in that position. I would even venture to accentuate the confession by saying that whatever you, my hearers, may be, he who addresses you is stone blind. I do not doubt that you will all recognise this, and I suspect, have not a little curiosity as to how I propose to fence with my ignorance. My purpose is to recognise it frankly and expose it openly to the light of day; so let us speak in chief measure of the many difficulties of the subject upon which you have asked me to address you.

Climatology and balneology are by no means identical in aim, nor shall I attempt to decide as to the relative importance of each; but from the point of view which I intend to take they may well be considered very much in common. As to the importance of climatic conditions, in their effect upon disease, I take it that we are all agreed. The difficulty is to say when and how these conditions act, and what is the effect in the given case. That the effect is very positive any number of illustrations might be given to show. Some years ago, in giving an address in the North of England, I introduced the subject of the fear of death, and contended that it had been greatly exaggerated because I had so seldom been asked the question, "Am I going to die?" by anyone who was gravely ill. After the meeting a physician of great experience, practising in the neighbourhood, said he did not agree with me—he was frequently asked the question, and he took it that the climate of the North of England produced more grit than did that of the South. In a leading article in the *Times* only the other day on the Mohmand rebellion, that some of the clans had fought better by far than others was attributed without question to the fact that the fighters had remained in the uplands; those who had gone soft had long lived in the plains. The Icelander requires tallow and blubber as necessary articles of food: we should have a difficulty in making both ends meet upon them. At Kimberley, I am told that glycosuria is very common amongst the obese. And this reminds me of another interesting fact I noticed many years ago, that whisky, although very largely indulged in, I believe, in Scotland, very seldom there leads to cirrhosis, whereas in England we know that it does so frequently; and it is said to have much to do in tropical climates with abscess of the liver. I ought to add, that, having made this statement years ago in print, and being subsequently asked for further information on the subject, I wrote to the late Sir William Gairdner telling him my experience and asking him if he could confirm it, and he replied that he believed my statement to be correct as regards the existence of cirrhosis in the North, but that (this, no doubt, with a twinkle in his eye) he thought that the reason might be that the "Scotch" up there was better than what we drank in England.

Talking of Scotland reminds me also of two other illustrations. Some few years ago a man came to me who had all the aspect of advanced malignant disease of the liver: he was deeply jaundiced, with a very large liver, which from its general characteristics left no doubt in my mind of the nature of the disease. His one cry was "Let me go to Scotland, and I shall get well." He was too ill, I considered, to go so far, but at last I gave way, if he would consent to take his doctor with him. This he agreed to do. He went to Scotland, speedily began to mend, and got well within a few weeks, and he has been well ever since. Do not suppose that I claim that the climate of Scotland can cure a cancer. The diagnosis was, of course, a mistake, and should have been, perhaps, that of gall-stone or pancreatitis; although I have always contended—and as it falls in with one of the main contentions of this address I may as well say it again—that we are far too mechanical in our doctrine of jaundice; there is many a case that is paralytic and not obstructive, functional that is, and not organic, and such a case the Highland air might well upon occasion turn towards health.

But how absolutely ignorant we are of the causes of such effects. We are all of us daily using the terms "bracing" and "relaxing"—we know what we mean as a subjective sensation, and we see the effect in the objective renovation. But what has been the cause? Take Brighton for example, because it so often affects people as to make them headachy, giddy, anorexic, rheumatic—bilious, in a word, as it is called. The climate is obviously of a peculiar character, but for those whom it suits there is no place more invigorating. I know of many places—you all know plenty more—good residential sites for the many, yet at which some who live there declare that when in residence every scrap of energy and *joie de vivre* has departed from them.

Take another large class of people, those whose intestinal action is of barometric type, may I call it. I mean those who are liable to diarrhœa at the onset of change of weather. A friend said to me the other day that I must not ask after his health when the wind was in the east; dear old Mr. Jarndyce is a representative of no uncommon experience. Think, too, of the extraordinary effect that a thunderstorm will have upon some nervous centres, reducing them to a state of absolute collapse. What may this not mean in the mutual relation between electrical energy and health?

Thus, with an advancing knowledge, the question of climate is not only intensely interesting, but is also potentially important, and lands us almost in the position that nothing, not even "water finding," is impossible.

And while I was considering how best to put before you this intricacy of our subject, there crossed my path a man well versed in the practical dealing with Caisson disease. You all know that in death from it —a climatological one, so to speak—the right heart is distended by free nitrogen, and the tissues are permeated with similar gas. It has been further found that stout people are more liable to be affected than the thin, and it is said that this is due to their greater capacity for holding up nitrogen in the tissues.

I do not suggest that in dealing with disease as influenced by climate we are ever called upon to deal with any such extreme conditions as are present in Caisson disease, but I do mean to say that what exists in the one case in extreme degree may quite possibly be present in many another case in imperceptible degree, and may thus initiate some minute alterations of nutrition and structure that by and by will produce disease. We all know that the opposite of Caisson disease, residence at extreme altitudes, will produce changes that more or less come under the head of disease, and may call for treatment in the ordinary course; and I see no real objection to the conjecture that some climates to others may be described as mildly caissonic, or that, in our attempts to treat disease, we must study the varying conditions and degrees of the interchange of gases under high and low atmospheric pressure of extremely moderate variation.

It is well recognised that the unbroken skin is a great bar to the absorption of drugs, and nothing is to be expected in this respect from an ordinary bath. Nevertheless, in the light of advancing knowledge the possibility, even in such cases, of very delicate reactions and interchanges needs to be considered. Certain it is that baths of various sorts are believed to effect very marked results. Many a man, for example, will take a daily warm bath in his own house with no appreciable effect, whereas if he takes one at Aix les Bains he may tell you that he became so depressed and enervated that it did him more harm than good. Needless to say, all such statements need careful sifting. Many things need to be considered besides the actual bath—the whole environment, atmosphere, temperature, diet, friends, doctor, even sugestion—all come in, and the exact share of each is hard indeed to estimate.

Again, there is some evidence that suggests that under the influence of electrical currents certain salts can be made to penetrate the skin and tissues. Mr. Moir told me that in the Blackwall Tunnel he noticed that the greater the contamination of the air with carbonic acid the more did Caisson disease threaten, and he ventures to suggest that although it has no demonstrable influence in the production of the disease, it may yet by its presence serve some such action as the so-called fluxes do amongst the metals.

Moreover, we have nowadays to consider the effects of the various modifications of heat, electricity, and light. I saw the other day a doctor who, with an irritable spot on his leg, apparently of no importance, had exposed it (as he thought with all due caution) on three or four occasions to the influence of $x$-rays; and slowly thereafter, many days intervening, there came a deep-seated destruction of the tissues of the leg that has kept him out of work for months. We are all, indeed, too familiar now, with those terrible cases of dermatitis that pass on to epithelioma under kindred circumstances, and have necessitated the removal of limbs. The growth of a cancer due to the repeated exposure of a part to the $x$-rays! Such a thing cannot but make one ponder, and ask oneself: "What has happened to produce so determined an effect?" And again, one may be forgiven for wandering on into the conjecture that what has happened in this case in so pronounced a manner may possibly give a clue to like causes in more diluted form, which, acting insensibly over far greater ranges of time, might result in similar disease, and which, on the other hand, recognised in their first state, might be corrected.

In this sense let me next say something having more specific reference to mineral waters in general, and our work at the watering places whence they come. I suppose that most people would admit it to be true that all such waters are more efficacious when taken at their source—perhaps, we might say, when drunk on the premises—than when sold in bottles. If this be true, what does it mean? Probably in some measure that the composition of the water alters by keeping; and there are infinite possibilities of this sort if one tries to think of them. No doubt there are gases in minute proportion in the natural water on draught that add to its efficacy, although as chemists we may not be able to recognise and take cognisance of them. Then I know not what the chemists may have to say about radium, but I fancy that radio-activity and all the new knowledge derived from that source may well have some bearing upon the potency of waters taken fresh. But at their source, or not, any one of the natural mineral waters has, over and above the one or two chief constituents, many others present in minute quantity, and who shall say that one and all in some subtle combination—some acting, perhaps, as intermediaries, as already suggested—are not potent to influence the habits or tendencies of our several organs, and in so doing, to influence the very beginnings of disease.

Let me illustrate what I mean by a reference to uric acid. It is clear that under a variety of circumstances, particularly as we arrive at and pass middle age, our tissues acquire the habit of producing materials of this sort—products of imperfect combustion we call them—in excess. Much as if, in a furnace under the influence of modified draught, now this, now that ash would be produced according to the circumstances dominant at the moment. We then become those to whom the doctor applies the much-scoffed-at term of gouty. "Everyone is gouty!" says the reproachful patient. Well, that true gout is rare indeed in comparison to the number said to be gouty must be admitted. But most people after fifty, some much earlier, arrive at that mildly decadent condition when their organs do not work so automatically towards the right, or so naturally make for health as is the case in earlier years, and the blood and juices become more or less charged with impurities, a condition which needs some generally descriptive term, and which waters in some way seem to help to dissipate.

And I can quite conceive that natural mineral waters of suitable kind may so impalpably, and yet definitely modify, say, the specific gravity of those juices, the storage of gases in the tissues, the activity of our regulatory nerves, the varied heat centres, the powers of our absorbents, or the circulation of the fluids, that the natural habit, that is health, might be revived. For, after all, what is health? what is disease in many instances but a habit of action on the part of the tissue? and fresh from their source, in all the virginity of their exit from the spring, one would suppose that the virtues of these waters must be readiest in power. And I may remark in passing that herein, perhaps, lies the reason that waters are seldom, as I think, needful to the young. Their lives make for health, if we watch without meddling; and so true do I hold this to be that I would hardly except the iron spas, which are certainly useful. So jealous am I to keep the young out of the range of valetudinarianism that even chalybeate waters only hold in my estimation the position of exceptions that prove the rule, that waters and baths are for the decline and fall, not for the rising decades of life. Nor are waters either, as a rule, for the very ill, who need the comforts and routine of home. A course of waters taken at their source mostly benefits the sound person who is out of health.

But there are other elements in a cure beside the drinking of the waters which all come into question when it becomes necessary to sum up the exact value of the waters themselves. The surroundings will in many cases have a large share in their success. It needs no saying that there is all the difference in the world between taking brimstone and treacle when dispensed by Mrs. Squeers and when mollified by such allurements as a skilled and elegant pharmacy is able to accomplish, between taking waters in bad weather or discomfort, or to the strains of music in beautiful surroundings, with all the sensuous stirrings of a fine summer, or its counterfeit, a well-warmed winter garden. Ah! there is the rub for us! If the English summer were but less fickle. The rays of the sun, its warmth, and fresh air are not things of the moment and of fashion as passing moods might seem to suggest; they are the everlasting sources of energy and the very springs of health. So that the more beautiful the surroundings, the more sunny the spot, the finer the weather, the more out of door the life, the more reposeful and pleasant the daily occupation, the more remote from the madding crowd, the better the results are likely to be.

Disease is too often so intimately the result of the interaction of our several organs and structures that it is not to be got out, unless its host is first broken on the wheel. The true meaning and pathology of uric acid, for example, will never be advanced by sending a man to a cure, and telling him that the gravel he there passes is a morbid material that is being washed out of him. Much nearer the truth would it be, to say that it is made on the premises. I have a fixed idea that in some persons undue water drinking disposes to the production of uric acid. Men go to these places in a condition of jaded vitality, and waters do not tend immediately to improve that condition; they may, indeed, and do, make them worse, as when, after a full course of waters, you end up most disappointingly with an attack of the very disease you have gone to rid yourself of. But it is all quite as it should be—the lethargic or choked organ necessarily turns out poor work, and the output for the time being gives evidence thereof. Nevertheless it is a stage in the process of recuperation. But when I go to a cure, so-called, I take no medicine save the water that I have come to drink, and the doctor has thus presented to him what is practically experiment after experiment, each carried out under as nearly as possible similar conditions; the only things that come to mar the accuracy of comparison being the attitude of the observer, and the individuality of the subject. These are important qualifications I admit, but it is no small advantage to obtain even this simplification and chance of precision, so much greater as it is than the common lot of the practitioner in ordinary, who has his thousand and one drugs to select from, and the necessity laid upon him to change them one after the other when the ill-success of one and another, or the nerves of his patient, seem to demand it.

Thus the skilled balneologist who keeps himself alert would seem to have a magnificent opportunity for adding to our knowledge and benefiting mankind. He is, as it were, a picket planted at the outposts of health, to report the early movements between it and disease. Here are to be seen the ebb and flow of the tide of life; and here, if anywhere, is the position to detect the early threatenings of morbid change; now, if at any time, is the moment to check the insidious advances of the invading enemy. The balneologist's true work then, as it seems to me, is to study physiology, not morbid anatomy, and therefore at watering places, if anywhere, should be gathered the most skilled and penetrating observation. Nothing must be allowed to escape us here. The air we breathe and exhale, the excretions of the skin, intestines, kidneys, liver, the expenditure of vital energy in every form, the supplies that are taken in for its renovation, all must be estimated in the most detailed way if any correct conclusions are to be drawn; and if so, can there be any doubt that the borderland of disease needs the keenest eye, the most alert mind, the imagination curbed to scientific uses, and reared, too, not in the good rich soil of striking new facts, but on the common, comparatively mean things, if any such there be, in the economy of life?

And yet, although all this is true, although there are those in our midst who work ever in this mind and spirit, how little addition to our knowledge of physiology or medicine has as yet come from any of these, our many, should be, seats of learning. But the reasons for this are not far to seek, and follow from what has gone before. In the first place the public resorts to a water cure with a but half serious purpose in many cases. Many never see a doctor while at the spa, and few come under any careful observation. Then the monotony of conditions, again and again repeated, tends, as one can readily understand, to envelop everyone in the shroud of treatment by routine. Nor is that all, for greater even than these is the inherent intricacy of the ground to be explored; that borderland upon the confines twixt health and disease, where deranged function passes into disease. Oh, for the insight that might detect the passing! Have you any doubt about it? If I educate my brain in faulty methods of action—if impulsive, and I encourage its impulsiveness; or restless, and I play upon its restlessness; lethargic, and I give way to excessive dormancy, I gradually create a bad habit, an unhealthy condition, that tends to increase until it is quite beyond my control, when it has become a disease. May I give this suggestion as an illustration?

I have often thought that the disease we know as disseminated or insular sclerosis—of the origin of which we know next to nothing—might conceivably start in those fits of dormant thought and suspense of will that are seen best in the hysterical woman, but which, in lesser degree, no doubt, have a very wide range of existence in all conscious nervous action. I can readily imagine that, as in parts of the lung, suspended respiration, if sufficiently prolonged, will lead to collapse, and then to induration, so areas of suspended action in the brain might eventually lead to atrophic and degenerative changes in those areas; eventually to capillary and connective tissue change, and these to the slow development of those curious nodules that characterise the disease. Thus it is conjecturally possible to trace it directly to functional inertia.

Such disorders of function in the case of the brain we call insanity, and we are not surprised when morbid anatomy detects no disease. But is it otherwise with other organs and tissues? It cannot be. The same law prevails everywhere: what a man sows, that shall he reap. But we are so much more familiar with the changes worked by disease in those other parts that we even sometimes permit ourselves to argue from thence that there can be no error of function that is not preceded by structural change. But no physiologist can question that in stereotyped aberration of function lie the beginnings of such structural changes; or that in its very earliest stage structural change is either non-existent or so imperceptible and infinitesimal in degree that it might be righted in a moment. We may call the disease by any name we please, whether it be pronounced change or subtle intoxication; but it is the functional failure that is earliest appreciable in many cases, and little by little that failure bites out the engraving that we call disease.

It is easy to say for the most part when there is such coarse change as tuberculosis or cancer, or this, that, or the other, that makes up the rough morbid anatomy of disease, but what we have still to find out and to tell is when a man is putting himself in the way of inviting these things to come, when function is becoming deranged and can yet be righted. We cannot cure a cancer or consumption—at least not yet, if ever. But we could cure even these, in all probability, if we could detect and alter the status, the *x* or other ray, that allows of their introduction, or goads them into being. That is what open air does for tubercle. There is no cure for consumption when it has got a firm hold, but he who harbours it will make short work of it on its first introduction if his powers are such and his environment such that they make for health.

The doctor who elects to plant himself at a mineral spring has chosen a very difficult position. A man may be at work for half a life and more, and find at the end, or, more probably, others will find for him, that he is off the line. Happy will he be if he can see that even so his energies have not been wasted, that by the poignant suggestiveness of his ill-success he has helped to establish others upon more productive ground, and thus steered us all for the promised land. But not thus does the natural man view the treatment of disease, not thus even the doctor himself when he comes to be sick, if we may for the moment distinguish between personal interest and scientific detachment. We put ourselves passively into the hands of our brother to be cured as we call it, but our duty is a much more strenuous matter if the average value of watering places, of the health of the future, is to be better than in the past, or what it might be. Co-operation between doctor and patient must, indeed, be our watchword, but everyone will need to think more about, and know more about himself—that is, how his own machine is working, not introspectively and morbidly, but naturally, as a wise motorist knows his motor; to recognise that the machine when out of order, "with its tricks and its manners," must be helped to right itself, and the observer by himself has, from his own observation in no small measure, to tell how the restoration comes.

To show how true all this is, one of the very latest approaches of scientific investigation towards the light—the opsonic dealing with disease—is in the direction of creating an aspiration or a want in the very lowest realms of our being towards this self preservation and self restoration. I know not what will be the final judgment upon the practical application of opsonic indices to the treatment of disease; I think that as regards its present utility it is not "quite, quite," as one lady said of another; but this, at any rate, will be, I think, always true, that the idea is a philosophical one in its conception, in that it is an attempt to eradicate disease by stimulating under observation and control what is supposed to be a natural physiological action.

And one may venture to surmise that it is in this very direction that mineral waters and climate do their work. I doubt much, if they wash out this, and purge away that, or that their chemical action, if chemical it may be called, is of any such clumsy sort. Rather would I think of them as full of a nascent activity, calming or stimulating here, altering or disintegrating there, in that perfect transformation scene of imperceptible metabolism that is still wrapped up in the mystery of living organisms, and which has been, in all ages, the inspiration of discovery. Such inanimate influences as these have, as we now know, boundless powers, as yet only on the threshold of recognition, but immeasurable as they seem to be, they are in all probability far below the possibilities foreshadowed in the energy of all animate existence.

Now, Sir, in thus dwelling upon questions of this kind, some, perhaps, may be inclined to ask, "What is the use of thus dreaming dreams?" But think not so! "Let there be light" is a good working ideal. It is no more than what this old world has worked up to through all its ages, since first it breathed into existence, and throbbed with the impulses of organic life, and age after age in turn has surely given back the only possible verdict, "And there was light." And as time goes ever onwards this must still be true.

